# TiO_2_ nanoparticles immobilized on mortar spheres as a strategy for efficient photocatalyst reuse: new UV reactor design for dye removal

**DOI:** 10.3389/fchem.2025.1581274

**Published:** 2025-04-28

**Authors:** J. R. Cob-Cantú, K. López-Velázquez, J. G. Ronderos-Lara, E. R. Hoil-Canul, C. Castillo-Quevedo, L. A. Maldonado-López, J. L. Cabellos-Quiroz

**Affiliations:** ^1^ Universidad Politécnica de Tapachula, Tapachula, México; ^2^ Secretaría de Ciencia, Humanidades, Tecnología e Innovación (SECIHTI)-Universidad Politécnica de Tapachula, Tapachula, México; ^3^ División de Ciencias Básicas e Ingeniería, Universidad Autónoma Metropolitana-Azcapotzalco, Ciudad de Mexico, Mexico; ^4^ Departamento de Fundamentos del Conocimiento, Centro Universitario del Norte, Universidad de Guadalajara, Guadalajara, Jalisco, Mexico; ^5^ Centro de Investigación y de Estudios Avanzados del Instituto Politécnico Nacional, Unidad Mérida, Mérida México

**Keywords:** catalyst recovery, dye degradation, durability, photocatalysis, water treatment

## Abstract

TiO_2_ nanoparticles were immobilized on mortar spheres and subsequently packed into a tubular reactor equipped with a concentrical submergible UV lamp for photocatalytic decolorization of aniline blue solution. The microstructure and chemical composition of TiO_2_ layer on the spheres, the efficiency for aniline decolorization, and the durability of the TiO_2_ coating were studied. In this work, the mean thickness of the TiO_2_ layer was 4.01 ± 0.55 µm, while the mean mass loading on the substrate was 5.6 ± 0.61 mg/cm^2^. Then, the photocatalytic reactor showed excellent performance for dye removal, reaching levels between 95%–97% in 150 min under UV light. Moreover, by radical scavenging experiments, *h* ^*+*^, O_2_^.-^, and ⋅OH were identified as the main reactive species. Even after twenty consecutive cycles, the removal efficiencies were higher than 83% and the decrease of efficiency was related to the partial detachment of the TiO_2_ layer (mean thickness decreased to 2.17 µm) which was verified by FESEM-EDX and metallographic microscopy. Finally, based on results, it is worth noting that the effective immobilization of TiO_2_ photocatalyst on the mortar spheres as substrate facilitates catalyst recovery, improves recyclability, and enables continuous water treatment. Therefore, this technology is a promising option for the removal of dyes in water, we even suggest that the proposed photocatalytic reactor could be scaled up for the treatment of effluents from textile industries, contributing to the abatement of water pollution.

## 1 Introduction

Due to the rapid development of industrialization and urbanization, the water pollution is increasing dramatically worldwide, mainly due to wastewater discharges from industries such as textile, leather dyeing, plastic, rubber, paper, photographic, cosmetic, drug and food processing, as well as landfill leachates (by hydrolysis of several compounds) and discharges from livestock, hospital and municipal activities (Aguilar-Aguilar et al., 2023; Azizi et al., 2022; Pedro-Cedillo et al., 2019). These effluents contain various types of persistent and toxic pollutants, including pharmaceuticals, illegal drugs, personal care products, pesticides, nutrients, solvents, plasticizers, brominated and chlorinated flame retardants, surfactants, hormones, microplastics, and synthetic organic dyes, among many other compounds (Aguilar-Aguilar et al., 2023; Rathi et al., 2021; Rathi et al., 2021). To treat these wastewater, numerous investigations have focused on alternatives for the effective removal of contaminants through various treatment techniques, including physical, chemical, and biological processes (Kumar et al., 2022). Among these technologies, heterogeneous photocatalysis with TiO_2_ has gained great attention due to its high activity in UV range, low cost, easy operation, good reusability, gentle reaction temperature, and high efficiency for complete degradation and mineralization of several contaminant mixtures (Feng et al., 2019). However, the main disadvantage of photocatalysis-TiO_2_ for the scale-up is the use of the catalyst in aqueous suspension since an extra filtration step is required to separate and reuse the TiO_2_ powders, which can be solved if the TiO_2_ is immobilized on an appropriate substrate to facilitate catalyst recovery, improve recyclability, and allow the water treatment in continuous cycles (Durán et al., 2018). In recent years, many kinds of substrates have been explored, including glass (Cunha et al., 2018; Malakootian et al., 2020), activated carbon (Gar Alalm et al., 2016; Zeng et al., 2021), polymeric membranes (Tran et al., 2020), chitosan microbeads (Aba Guevara et al., 2021), cellulose (Hamad et al., 2018), stainless steel (Monteserín et al., 2024), clay (Bunea et al., 2023; Sraw et al., 2018), quartz sand (Eddy et al., 2015), ceramics (Danfá et al., 2021), and others, such as cement paste and mortars (Choi, 2024; Feng et al., 2019). Cement-based materials are one of the most widely used and consumed materials worldwide, and the plentiful pore structure, excellent chemical stability, durability and low-cost make cement-based materials as a suitable substrate for photocatalyst immobilization. In previous studies, TiO_2_ has been immobilized on cement substrates by different methods: mixing TiO_2_ with the cement powder, penetration of TiO_2_ slurry into cement blocks, and sprinkling TiO_2_ powders on the surface of fresh cement. However, these methods have shortcomings such as low photocatalytic activity and less uniform dispersion (Feng et al., 2019). Therefore, it is necessary to explore other techniques that can be implemented to remove dyes more effectively. Thus, in this research, TiO_2_ nanoparticles were effectively immobilized on mortar spheres using an easy-to-apply painting coating. In this sense, it is worth noting that the effective immobilization of TiO_2_ on the mortar spheres can facilitate catalyst recovery and improve photocatalyst recyclability.

On the other hand, in most photoreactors the light source is located outside the solution and only a fraction of the irradiated light interacts with the photocatalysts. To overcome this problem, we have designed a tubular vertical reactor equipped with a submersible UV lamp inside and downward water recirculation. Then, the coated spheres were packed around the lamp to maximize the illumination of the photocatalytic surface and the generation of reactive species. Thus, the constructed reactor was assessed for the complete decolorization of an aniline blue solution, which was used as an example of organic pollutant from the textile industries. To our knowledge, a similar reactor configuration filled with TiO_2_-coated mortar spheres has not been reported yet. Therefore, the main objective of this work was to provide an effective and low-cost alternative to immobilize TiO_2_ nanoparticles on mortar spheres to facilitate catalyst recovery and improve photocatalyst recyclability during continuous water treatment cycles. The TiO_2_-coated mortar spheres were activated with UV light inside the vertical reactor as an alternative for water decontamination, and the microstructure, chemical composition, durability of the TiO_2_ coating as well as its efficiency in removing aniline blue were also studied.

## 2 Experimental section

### 2.1 Immobilization of TiO_2_ on mortar spheres

Commercial TiO_2_ nanoparticles (P25 Evonik Aeroxide) were selected for immobilization on the mortar spheres due to its high efficiency in many photocatalytic applications (Tobaldi et al., 2014). This semiconductor is made of anatase and rutile phases (percentage 80:20 approximately) with particle size distribution between 14 and 21 nm (Evonik Industries AG, 2023) and specific surface area between 35 and 65 m^2^/g. Furthermore, the UV-vis spectra of the TiO_2_ nanoparticles were recorded using Genesys 10S equipment (Thermo Scientific) from 200 to 800 nm, and the optical band gap of the catalyst was calculated by the Tauc plot method (Guayaquil-Sosa et al., 2017).

Mortar spheres were made by mixing ordinary Portland Cement (CPC 30R RS, Cruz Azul, Mexico), river sand from local suppliers (size particle < 1 mm) and tap water in ratios 2:1:1 w/w, respectively. The mortar spheres were manufactured in plastic moulds (diameter of 2.5 cm) with a setting time of 24 h, washed with plenty of water to remove any impurities, and dried for 24 h at room temperature, it is important to mention that the average mass of the mortar spheres was 18.47 g and its density was 2.25 g/cm^3^ (data calculated for 30 dried spheres).

For the immobilization of the photocatalyst on the mortar spheres, a TiO_2_ suspension was prepared by mixing 5g of powder in 50 mL of ethanol (96%) and placed 15 min in ultrasound water bath (70 Hz) for a complete homogenization. Immediately, TiO_2_ photocatalysts was supported on the mortar spheres by the painting coating technique using a soft paint brush (width 0.7 cm) alternating the direction of three applications, then the spheres were dried at 60°C (1 h) between each layer. After 24 h of drying at 60°C, the spheres were washed with distilled water to remove the excess of ethanol and TiO_2_ that did not adhere to the surface. To study the effective immobilization of TiO_2_ on the substrate, several spheres were randomly selected and analysed by FESEM-EDX technique (JSM-70601F, JEOL) and FTIR-ATR spectroscopy from 4,000 to 600 cm^-1^ (Nicolet iS50, Thermo Scientific). The thickness of TiO_2_ coating was measured by metallographic microscopy (Axiolab 5, Zeiss) and the mass of the catalyst supported on the spheres was estimated by a gravimetric method.

### 2.2 Reactor setup

**FIGURE 1:**
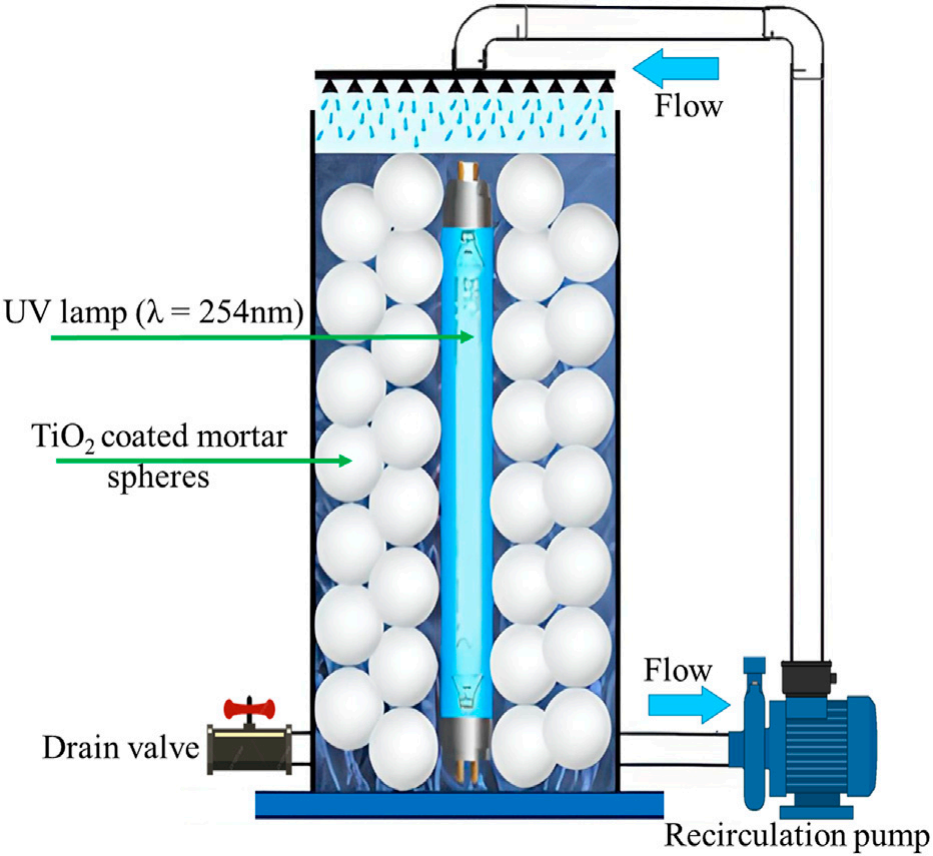
Schematic configuration of the photocatalytic reactor packed with TiO_2_-coated mortar spheres.

For the construction of the reactor, a transparent acrylic tube was employed (diameter 10 cm, length 35 cm, 5 mm thick). Then, a submersible UVC lamp (7W, 110 V, λ = 254 nm, Pyhodi) was installed into the reactor, similar to those reported by Durán et al. (2018), for aniline degradation; a recirculation pump (10 L/min, 12 V) and a drain valve were also installed as shown in Figure 1. It should be noted that the reactor was operated with downward recirculation and a water diffuser was employed to generate a turbulent flow within the reactor to promote the interaction between the contaminant and the photocatalytic surface. Finally, the reactor was packed with sixty TiO_2_-coated mortar spheres which were manually arranged around the UV lamp.

### 2.3 Photocatalytic tests

In this study, aniline blue (C_32_H_25_N_3_O_9_S_3_Na_2_, CAS 28631–66-5) was utilized to simulate pollutants in water since this compound is an important raw material of dyes, and is always a common by-product from the azo dye degradation; aniline have been listed as priority pollutant and is difficult to be completely removed in dyeing wastewater treatment (Zhang et al., 2021).

The experiments for the photocatalytic decolorization of aniline blue were performed at natural pH (6.0–6.6), atmospheric pressure and room temperature, recirculation at 10 L/min, and UV light as illumination source (λ = 254 nm). The reactor was operated in batch mode to treat 1.2 L of aniline blue solution (at initial concentrations of 5, 10 and 15 mg/L) using tap water and distilled water as aqueous matrix, the main characteristics of tap water and distilled water are shown in Table 1, it is worth noting that the temperature of dye solution during the experiments was 27.0°C ± 0.9°C. Before the UV irradiation, the aniline blue solution was stirred by recirculating into the reactor (30 min in the dark) to ensure the adsorption-desorption equilibrium among the dye and the supported TiO_2_. Then, the UV lamp was turned on and several water samples (5 mL) were taken every 30 min up to 150 min for subsequent analysis; moreover, the samples were centrifuged (4,000 rpm × 5 min, GP6 Scientific Centurion) to remove possible TiO_2_ particles and the concentration of aniline blue was measured by UV-Vis spectrophotometry at max wavelength absorption λ = 630 nm (Genesys 10S). The effect of initial dye concentration was investigated and the efficiency in terms of dye removal (DR) was evaluated as follows: %DR = ((C_0_ - C_t_) × 100)/C_0_, where C_0_ and C_t_ (in mg/L) are the initial dye concentration and at time t.

**TABLE 1:** Main characteristics of the tap water and distilled water used in the photocatalytic experiments for dye removal.

Parameters	Tap water	Distilled water
pH	6.4	6.0
Dissolved oxygen (mg/L)	5.9	4.3
Electrical conductivity (µS/cm)	436.0	0.4
Total dissolved solids (mg/L)	275.2	ND
Turbidity (NTU)	<1	ND
Total suspended solids (mg/L)	ND	ND
Temperature (°C)	27.7	27.0
Chemical Oxygen Demand (mg/L)	15.0	ND
Alkalinity (as mg/L of CaCO3)	182.0	ND
Total hardness (as mg/L of CaCO3)	142.0	ND
Sulfite (mg/L)	10.0	ND
Phosphate (mg/L)	0.3	ND
Nitrate (mg/L)	ND	ND
Chloride (mg/L)	40.0	ND
Iron (mg/L)	ND	ND

Data is presented as an average of three measurements. ND, no detected.

## 3 Results and discussion

### 3.1 Characterization of the TiO_2_ layer on mortar spheres

**FIGURE 2:**
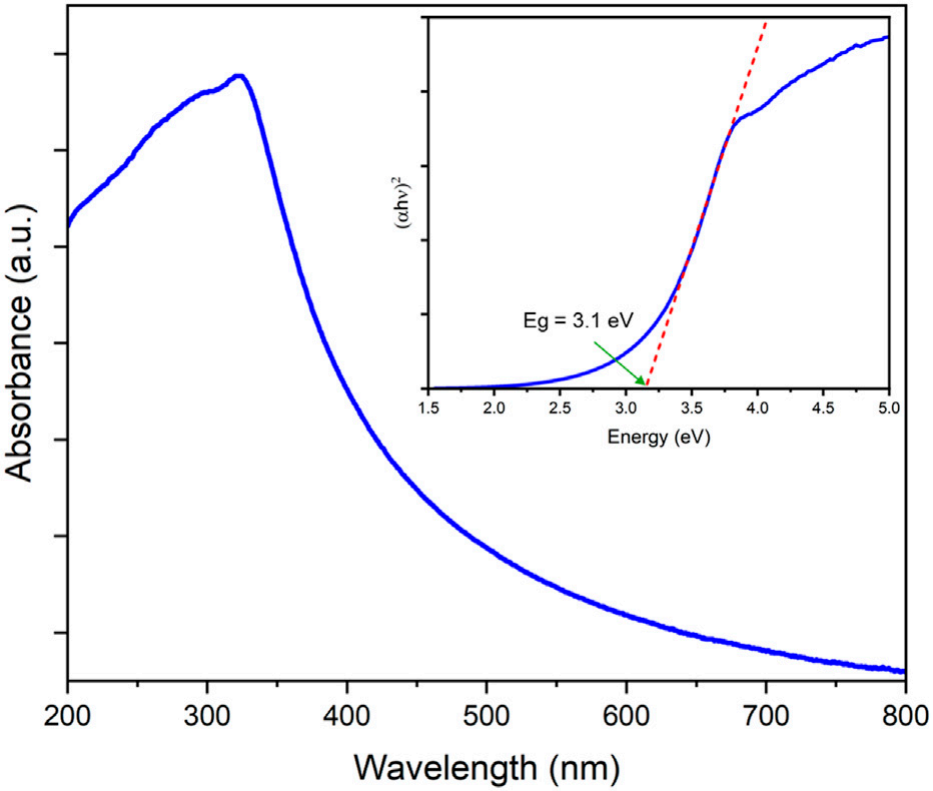
UV-Vis spectra of the TiO_2_ nanoparticles. Inset: optical bandgap calculated by the Tauc plot.

Prior to the immobilization of the TiO_2_ nanoparticles on mortar spheres, the UV-Vis spectra absorption of the catalyst was investigated; Figure 2 shows the UV-Vis spectra recorded from 200 to 800 nm and the λ_max_ was observed at ∼322 nm, which reveal its excellent UV absorption capability as has been widely reported (Galata et al., 2019; Guayaquil-Sosa et al., 2017). Moreover, the optical bandgap of the TiO_2_ nanoparticles was calculated from the recorded UV-Vis spectra using the Tauc method and a value of 3.1 eV was obtained, which is in agreement with the bandgap reported for commercial TiO_2_ P25 Evonik, between 3.1 and 3.3 eV (Guayaquil-Sosa et al., 2017; Pennington et al., 2018; Siah et al., 2016).

**FIGURE 3:**
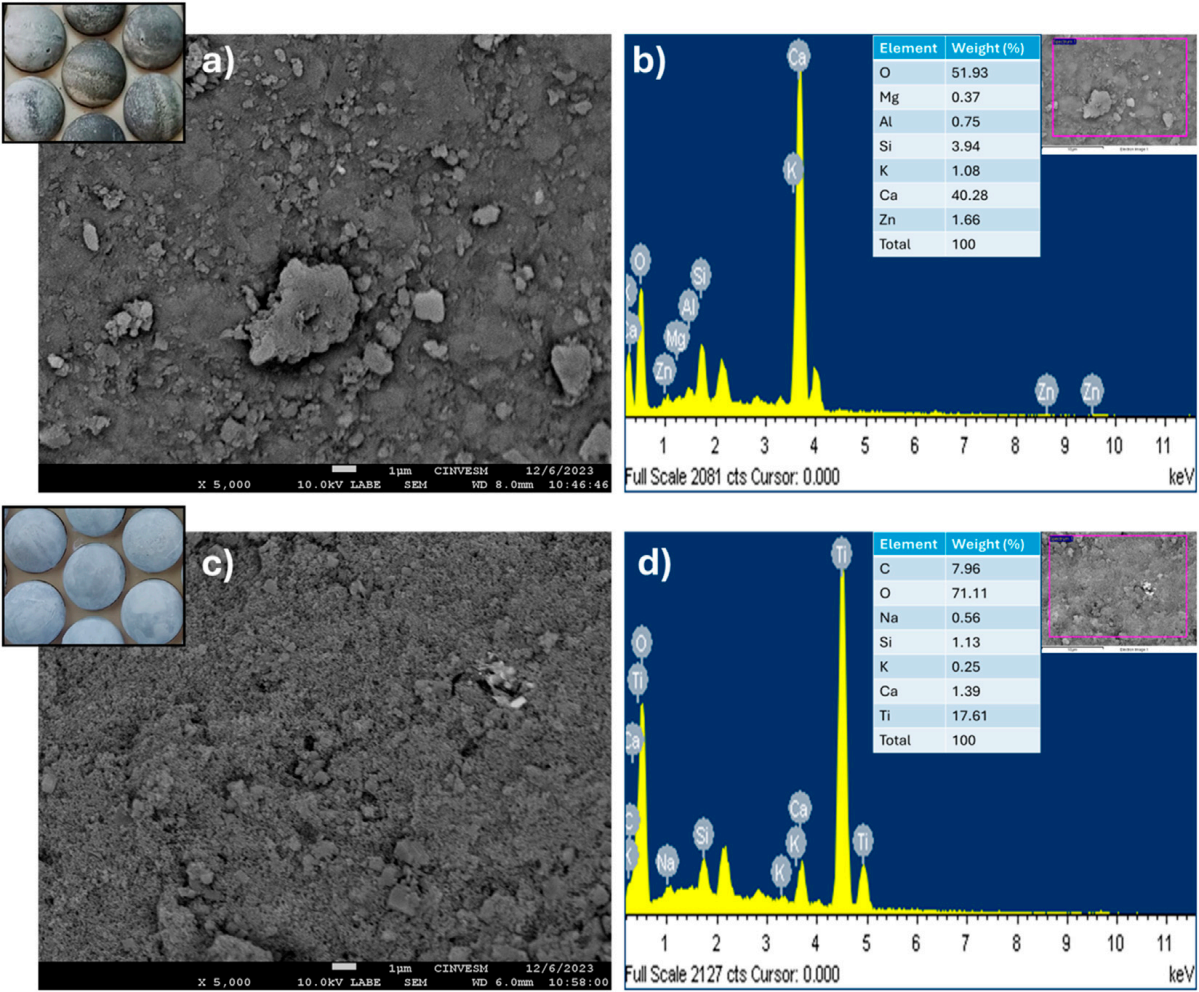
SEM image and EDX analysis for **(a, b)** surface of a mortar sphere without TiO_2_, **(c, d)** TiO_2_-coated mortar sphere.

Prior to the photocatalytic applications, the morphology of the TiO_2_-coated mortar spheres was investigated by FESEM technique and selected representative images are shown in Figure 3. At the same time, EDX analysis was used to estimate the surface chemical composition of the studied spheres with and without TiO_2_. Figure 3a shows the surface of the uncoated TiO_2_-mortar spheres, showing predominant heterogenous and grainy surface, which was considered as a good support for TiO_2_ nanostructures according to Gholami et al., (Gholami et al., 2018). Moreover, EDX analysis revealed that the surface of the spheres is composed of elements characteristic of cement, such as Ca, Si, and O (derived from CaO and SiO). From Figures 3b,c, it is clearly observed that TiO_2_ nanoparticles are uniformly coated on the substrate and randomly distributed in the grainy surface (Jafari and Afshar, 2016), while the main elements are Ti and O (from TiO_2_), C (probably from residual ethanol or CO_2_ absorbed on the surface), Si and Ca (from CaO, SiO, and interaction with the mortar surface).

**FIGURE 4:**
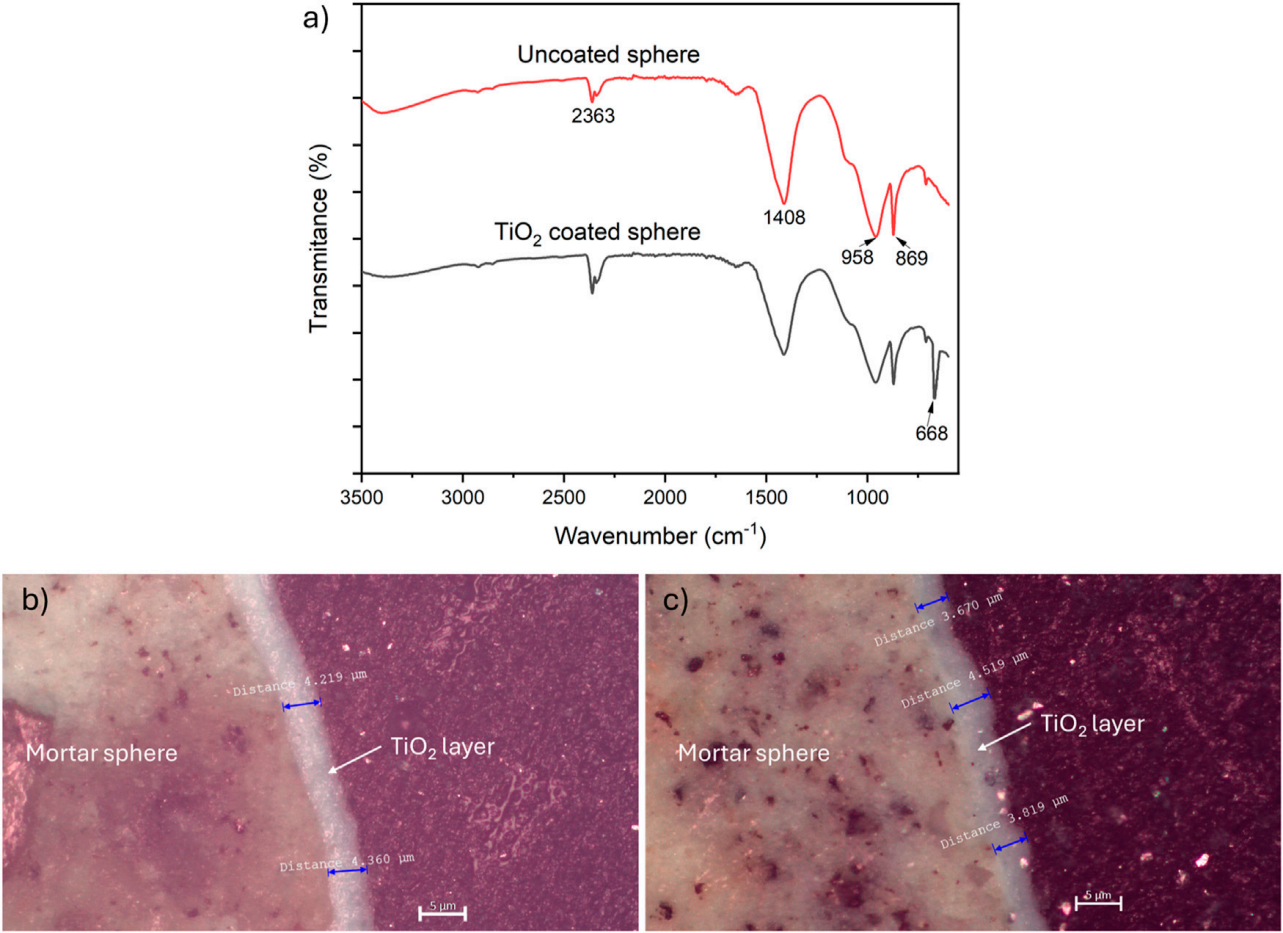
**(a)** FTIR spectra of the uncoated and TiO_2_-coated spheres. **(b, c)** Estimation of the thickness of TiO_2_ layer immobilized on the substrate, micrographs correspond to cross section of representative mortar spheres (metallography microscopy 10X).

Furthermore, the incorporation of TiO_2_ on the substrate also was investigated by FTIR-ATR technique and the results are depicted in Figure 4a, both FTIR spectra showed similar patterns and bands at 3,400 cm^-1^ were associated to the O-H stretching mode of water molecules absorbed on the surface of the spheres, the peaks around 2,363 cm^-1^ are often attributed to atmospheric CO_2_ molecules, while peaks at 1,408, 869 and 712 cm^-1^ corresponded to the vibration of CO_3_^2-^, as well as peaks located at 958 cm^-1^ corresponds to Si-O bonds from the mortar substrate (Meng et al., 2022; Ulukaya et al., 2017). It should be noted that the peak located at 668 cm^-1^ corresponds to the vibration of Ti-O-Ti bonds (Zhang et al., 2019) which confirms the successfully incorporation of TiO_2_ nanoparticles on the mortar substrate. On the other hand, several cross section of the TiO_2_ layer on the substrate were observed by metallographic microscopy (10x) and the mean thickness of the immobilized TiO_2_ coating was estimated to be 4.01 ± 0.55 µm, as can be seen in Figure 4. Finally, by the constant mass gravimetric method, the catalyst loading on the mortar spheres was determined to be 5.6 ± 0.61 mg/cm^2^, almost 3.5 times the mass loading on quartz surface used by Durán et al. (2018) for aniline removal.

### 3.2 Photocatalytic tests

**FIGURE 5:**
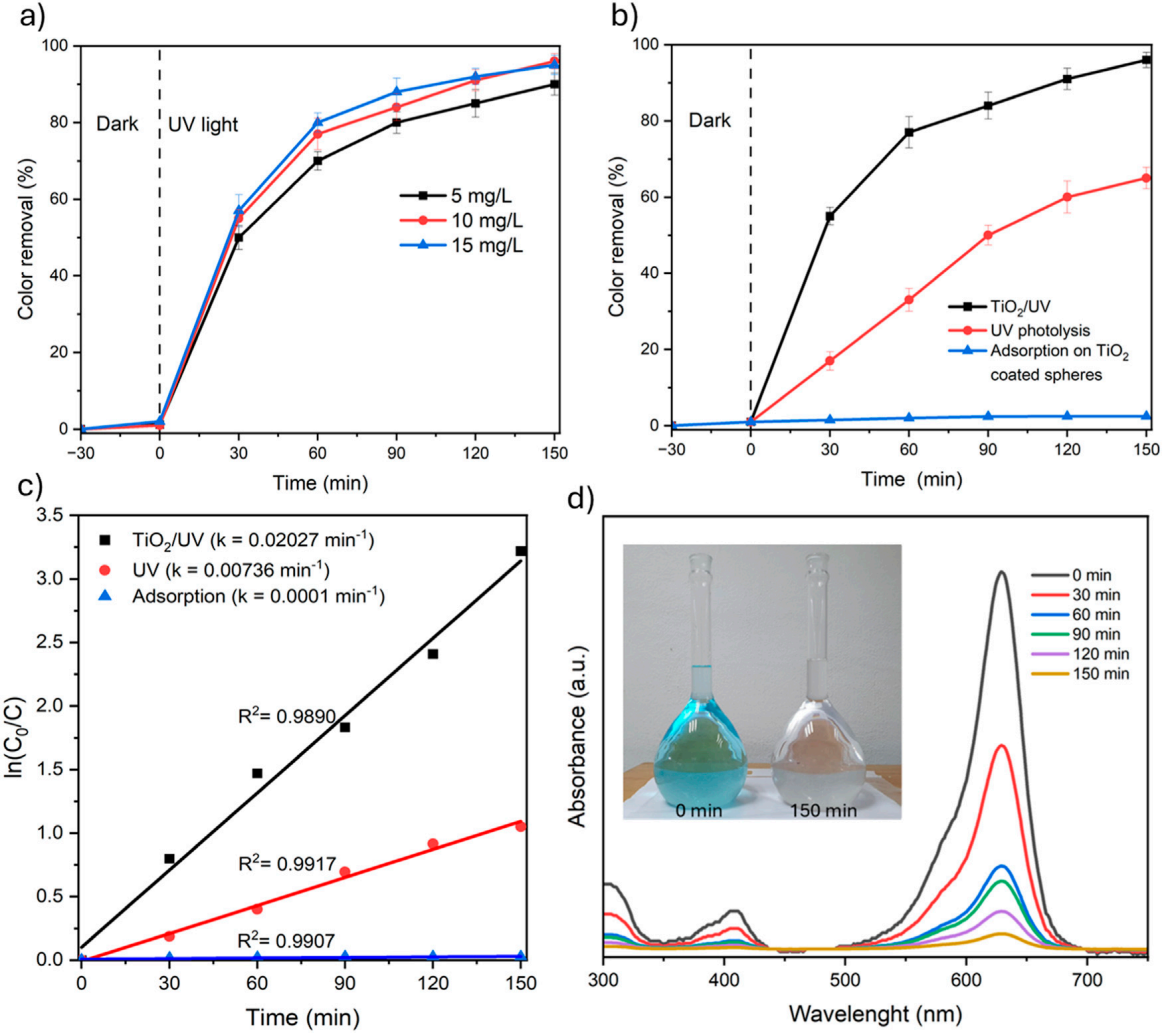
**(a)** Photocatalytic decolorization of aniline blue at different initial concentrations (5, 10 and 15 mg/L), **(b)** dye removal and control tests, **(c)** pseudo first-order kinetics modelling (*k*, min^-1^), **(d)** UV-Vis absorption spectra of aniline blue solution during the photocatalytic experiments.

The photocatalytic performance of the reactor packed with TiO_2_-coated mortar spheres (depicted in Figure 1) was assessed for decolorization of aniline blue solution under UV light (1.2 L in all cases), and results are shown in Figure 5. First, the effect of initial aniline concentration was evaluated from 0 to 150 min (Figure 5a) and slight variations for dye removal were observed; the efficiencies for dye removal were 90.3% at initial concentration of 5 mg/L, 96.3% at 10 mg/L, and 95.6% at 15 mg/L. In these experiments, the lowest efficiency (90.3%) was obtained at initial concentration of 5 mg/L, which could be related to the less interaction between the dye and the active sites of TiO_2_ and generated reactive species. Nevertheless, in this work, no differences for dye removal were observed at the highest concentrations of 10 and 15 mg/L. Previously, it has been reported that there is a significant decrease in the efficiency with increasing dye concentration, since more organic molecules (dye or by-products) are adsorbed on the active sites of TiO_2_ and the generation of radicals is suppressed, in addition, the high dye concentration can absorb a fraction of UV light and less photon´s energy reaches the active sites on the catalyst, resulting in a lower amount of reactive species (Abdellah et al., 2018; Feng et al., 2019). Based on the results, the increase of dye concentration did not affect the reactor efficiency, showing excellent performance for aniline removal at initial levels of 10 and 15 mg/L with efficiencies higher than 95%. On the other hand, in several investigations about photocatalysis with TiO_2_, distilled water is often used to prepare synthetic samples to obtain optimal conditions and the effect of the main constituents of water are underestimated. However, in this work, tap water was used as aqueous matrix in all experiments, and no interferences attributed to the content of TDS, COD, alkalinity, total hardness or chloride, among others, were identified with respect to the experiments conducted with distilled water.

In addition, control tests were conducted to explore the influence of UV photolysis and adsorption processes on aniline decolorization. Both experiments were carried out by triplicate, initial concentration of 10 mg/L, recirculation at 10 L/min, and 150 min of reaction. UV photolysis experiments were carried out using mortar spheres without TiO_2_, and the adsorption tests were performed using the uncoated and TiO_2_-coated spheres with the UV lamp off. In this sense, adsorption of aniline blue was not observed in the experiments using uncoated spheres, while the adsorption on TiO_2_-coated spheres was less than 3% (as depicted in Figure 5b). Moreover, UV photolysis contributes significantly to the dye removal (about 60%) due to the high photon´s energy (4.8 eV) that can degrade the dye molecules in solution and induce the dissociation of water molecules into ·OH. However, the best results were obtained by the activation of TiO_2_ under UV light to lead the production of reactive species such as ·OH, O2·^-^, *h* ^+^, and H_2_O_2_ (Feng et al., 2019), which increased the aniline blue decomposition rates 2.75 times compared with UV photolysis according to pseudo first-order kinetics displayed in Figure 5c, where the rate constant for the reaction kinetics (*k*) were 20.27 × 10^−3 ^min^-1^ for TiO_2_/UV photocatalysis >7.36 × 10^−3 ^min^-1^ for UV photolysis >0.1 × 10^−3 ^min^-1^ for adsorption on TiO_2_-coated spheres, it should be noted that the reaction kinetics of dye removal was well fitted for a pseudo-first-order reaction with *R* ^2^ > 0.9890. Furthermore, Figure 5d shows the UV-Vis absorption spectra of aniline blue at different reaction times and a marked decrease in the absorption levels was observed (between 300 and 750 nm), which is related to the decomposition of aniline blue and the decolorization of the water samples as can be seen in the inset (Figure 5d) where total decolorization of the aqueous solution was achieved after 150 min, showing the excellent performance of the photocatalytic reactor packed with TiO_2_-coated mortar spheres.

As comparisons with other studies, the immobilization of TiO_2_ on mortar blocks was evaluated by spraying method for the degradation of methyl orange under UV light and 73.8% of efficiency was achieved in 120 min (Guo et al., 2020). Also, TiO_2_ nanoparticles were supported by smear method on discs of cement paste and mortar (10 cm of diameter) for complete removal of rhodamine B, methylene blue and methyl orange under UV light in 50 min (Feng et al., 2019). Likewise, Gholami et al., reported the immobilization of TiO_2_ nanoparticles on the surface of fixed-bed reactor for removal of 98% of the chemical oxygen demand from wastewater after 8 h under UV irradiation (Gholami et al., 2018). Thus, it is evidenced that the immobilization of TiO_2_ nanoparticles on cement substrates is an effective alternative for photocatalytic water decontamination. On the other hand, substrates such as borosilicate glass spheres (Ø = 5 mm) and quartz rotating drums also has been employed for the immobilization of TiO_2_, achieving removal efficiencies between 89.6% and 96% for methylene blue and aniline respectively (da Cunha et al., 2016; Durán et al., 2018). In addition, Table 2 summarizes the comparison of the aniline blue removal efficiencies using different photocatalytic processes, this table provide current and important information to enrich the existing knowledge on the removal of aniline blue and other toxic dyes by heterogeneous photocatalysis.

**TABLE 2:** Comparison of different methods for the photocatalytic degradation of aniline blue.

Method	Aniline blue concentration	Experimental conditions	Time (min)	Degradation (%)	References
TiO_2_ photocatalysis	10 mg/L	TiO_2_-coated mortar spheres activated under UV light (254 nm)	150	96.3	This study
TiO_2_ photocatalysis	1 mM	Stainless-steel foams covered with TiO_2_ under visible light, pH 3, aerated	120	95	Vásquez et al. (2020)
NiO/CuO photocatalysis	1,000 mg/L	NiO/CuO nanocomposites activated under sunlight	60	91	Muhambihai et al. (2020)
CuO photocatalysis	3.75 mg/L	CuO nanoparticles (prepared by a green method) activated under sunlight	120	91.1	Mishra et al. (2024)
CoFe_2_O_4_/g-C_3_N_4_/bentonite nanocomposites as photocatalyst	10 mg/L	Photocatalysts were activated under solar irradiation in presence of H_2_O_2_	50	88.5	Thakurata et al. (2022)
Ag nanoparticles as catalyst	1 mM	Ag nanoparticles were prepared by a green method and employed for the photocatalytic degradation of aniline blue	12	86	Gokul Eswaran et al. (2023)
BiPO_4_ photocatalyst	20 mg/L	BiPO_4_ photocatalyst was evaluated for aniline blue degradation under UV irradiation (254 nm)	180	70	Azzam et al. (2019)

### 3.3 Photocatalytic mechanism and reusability tests

**FIGURE 6:**
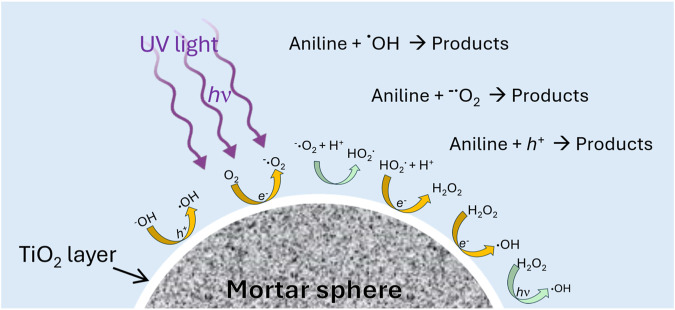
Schematic illustration for photocatalysis mechanism of TiO_2_ immobilized on the substrate.

Figure 6 shows the suggested photocatalytic mechanism for TiO_2_-coated mortar spheres, where the main reactions for production of reactive species are displayed. In this sense, TiO_2_ is a typical wide bandgap semiconductor (3.1–3.2 eV) with a well-described energy band structure (Guo et al., 2020) consisting of a valence band (VB) filled with electrons and an empty conduction band (CB). In this investigation, it is suggested that the irradiation energy from UV lamp (4.8 eV) higher than the bandgap of immobilized TiO_2_ promoted the excitation of electrons (*e* ^*-*^) from VB to the CB, and the corresponding holes (*h* ^*+*^) are generated in the VB, giving rise to “electron-hole” pairs. Then, electrons in the CB can further react with the dissolved oxygen in the aqueous medium and produce the superoxide radicals (O_2_^.-^). Moreover, the superoxide radicals can further react with hydrogen ions (H^+^) in water and generate HO_2,_ which can subsequently react with *e* ^*-*^ and H^+^ to produce H_2_O_2_. Finally, H_2_O_2_ can react with *e* ^*-*^ to produce ^.^ OH and ^−^OH, while the photocatalytic decomposition of H_2_O_2_ also contributes to generation of ⋅OH as reported by Gholami et al., (Gholami et al., 2018). On the other hand, the holes produced in the VB can capture ^−^OH ions from water to generate ^.^ OH. Therefore, according to those reported by several authors, *h* ^*+*^, O_2_^.-^, ^.^OH and H_2_O_2_ are the main reactive oxygen species that can degrade pollutants to small green molecules such as CO_2_ and H_2_O (Feng et al., 2019; Guo et al., 2020). In fact, in this work the reactive oxygen species were determined by radical scavenging experiments, where formic acid (FA), p-benzoquinone (BQ), and isopropanol (IPA) were added separately (1 mM each) and used as radical trapping agents of *h* ^*+*^, O_2_^.-^, and ⋅OH. The results evidenced that the dye removal ranged between 95%–96% without scavengers, while when FA, BQ, and IPA were added to the reaction, the dye removal decreased significantly, to levels of 14.5, 22.0, and 12.5%, respectively. Therefore, these experiments demonstrated that *h* ^*+*^, O_2_^.-^, and ^.^ OH were the main reactive species, responsible of the complete decolorization of aniline blue solution as can be seen Figure 5d.

**FIGURE 7:**
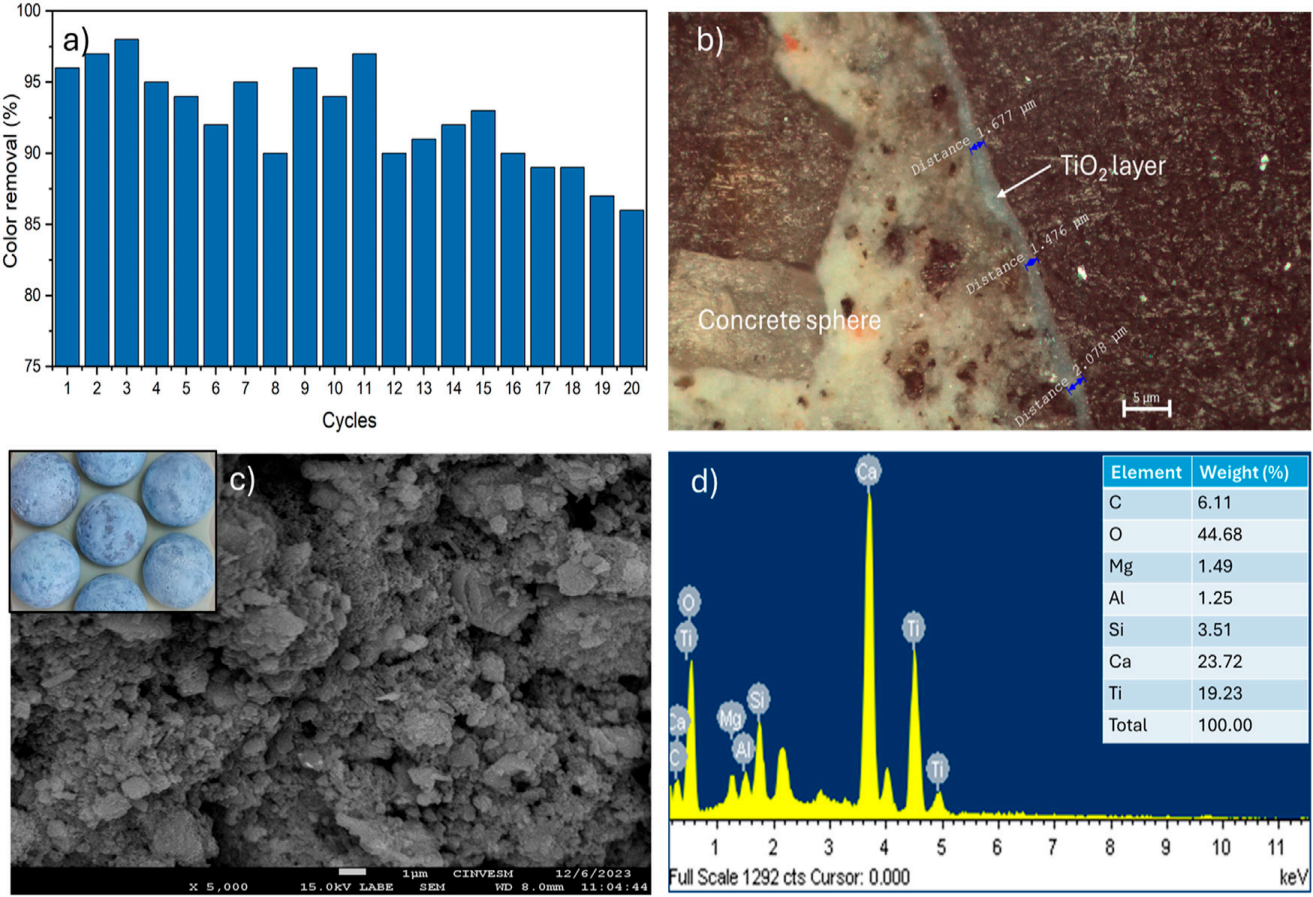
**(a)** results of reusability test (n = 20 cycles), **(b)** thickness of TiO_2_ layer after 20 cycles, **(c, d)** FESEM-EDX analysis of TiO_2_ layer after 20 consecutive reuses.

The study of the reuse and durability of catalysts is of great importance to reduce costs associated with this process for possible industrial applications. Therefore, in order to test the durability of the TiO_2_ immobilized on the substrate, the reactor was reused for twenty sequencing cycles under the best selected conditions (10 mg/L as initial aniline blue concentration, UV irradiation, recirculation at 10 L/min, pH 6.5–7.0, 150 min reaction time). After the reusability tests, ten spheres were randomly selected and the TiO_2_ layer was analyzed by FESEM-EDX and metallographic microscopy to explore the microstructure of the coating. Based on the results, the photocatalytic reactor proposed in this work shows good performance for dye removal during twenty consecutive cycles ranging from 83% to 97%, which is displayed in Figure 7a. However, in cycles 3, 7, 9 and 11, an increase in the performance of the photocatalytic reactor is observed, which can be attributed to variations inherent of the experiments, which even increases the reliability of the results. These variations during the reuse cycles were attributed to the changes in the composition of tap water samples, which may contain various salts, ions or organic compounds that can enhance or suppress the reactor efficiency over time. The efficiency for dye removal clearly decreased with the continuous reuse of the coated spheres, which can probably be related with the poisoning, photocorrosion or photodissolution of the catalyst as mentioned by López-Velázquez et al., (López-velázquez et al., 2023). In fact, by metallographic microscopy the decrease of thickness of TiO_2_ layer was verified, after twenty reuse cycles, the mean thickness of the catalyst layer decreased from 4.01 ± 0.55 to 2.17 ± 0.38 µm (Figure 7b). Moreover, the FESEM analysis of the reused spheres showed a heterogenous and grainy surface that evidenced the partial degradation of the TiO_2_ layer (Figure 7c) and coincided with the decrease of Ti content, as well as the increment of Ca and Si (from mortar sphere) determined by EDX technique (Figure 7d).

### 3.4 Limitations and prospects

This study constitutes a proposal of low-cost alternative for the immobilization of TiO_2_ nanoparticles on mortar spheres which were packed into a vertical UV reactor and excellent performance for decolorization of aniline blue was demonstrated. However, after twenty cycles of consecutive reuses the efficiency decreased from 96% to 83%, which can be related to the partial detachment of the TiO_2_ layer. Therefore, some improvements for the most effective immobilization of the catalyst can be investigated in further studies. For example, various mass ratios of the mortar components can be explored to modify the porosity of the substrate to improve the adhesion of TiO_2_ nanoparticles. Furthermore, some additives can be added to the TiO_2_ slurry to increase de adhesion of the catalyst to the surface of the mortar spheres, such as: waterproof emulsions (Feng et al., 2019), methanol (Gholami et al., 2018), 2-propanol or polyethylene glycol (Cunha et al., 2018). Even dip, spray or smear coating processes can be evaluated to improve TiO_2_ immobilization on mortar spheres.

On the other hand, as mentioned above, in this work the experiments were performed using a synthetic solution of aniline blue in tap water (10 mg/L). However, it is of great importance to evaluate the effectiveness of the photocatalytic reactor to degrade organic matter contained in real wastewater samples. Based on the results for photocatalytic decolorization of aniline blue, the proposed photocatalytic reactor can be a promising option that could be employed for industrial applications, mainly for decolorization of the effluents from the textile industries that contain high levels of organic dyes and many other organic components. However, the effectiveness of the proposed photocatalytic reactor for the removal of several cationic and anionic dyes, or mixtures of them, should be investigated in further studies. Finally, based on our experience with photocatalytic processes for water treatment and according with previous works (Abdellah et al., 2018; Cunha et al., 2018; Durán et al., 2018; Feng et al., 2019; Gholami et al., 2018; Guo et al., 2020), we suggest the use and application of commercial TiO_2_ nanoparticles in photocatalytic processes for water decontamination due to the wide advantages, such as: well-known physical and chemical characteristics, high photocatalytic activity under UV irradiation, structural stability, low cost, and among many others.

## 4 Conclusion

In summary, TiO_2_ nanoparticles were adequately immobilized on mortar spheres by the painting coating method which is an effective strategy for photocatalytic purposes. In fact, it is worth noting that the effective immobilization of TiO_2_ photocatalyst on the mortar spheres is an efficient strategy to facilitate catalyst recovery, improve recyclability, and enable continuous water treatment. In this work, the mean thickness of the TiO_2_ layer was 4.01 µm, while the mean mass loading on the substrate was 5.6 mg/cm^2^. Then, the photocatalytic reactor showed excellent performance under UV irradiation for dye removal reaching levels between 95%–97%. Even after twenty consecutive cycles the efficiencies were higher than 83% and the decrease was related to the partial detachment of the TiO_2_ layer which was verified by FESEM-EDX and metallographic microscopy. Finally, this technology is a promising option for the treatment of water contaminated with organic dyes, we even suggest that the photocatalytic reactor could be optimized and used on a large-scale for decolorization of effluents from textile industries, contributing to the abatement of water pollution.

## Data Availability

The original contributions presented in the study are included in the article/supplementary material, further inquiries can be directed to the corresponding author.

## References

[B1] Aba Guevara C. G. Sanjuan Galindo R. Gracia Pinilla M. A. Martínez Vargas D. X. Ramos Delgado N. A. (2021). Water disinfection using chitosan microbeads with N-C-C-N/TiO2 By photocatalysis under visible light. Top. Catal. 64 (1–2), 142–154. 10.1007/s11244-020-01356-2

[B2] Abdellah M. H. Nosier S. A. El-Shazly A. H. Mubarak A. A. (2018). Photocatalytic decolorization of methylene blue using TiO2/UV system enhanced by air sparging. Alexandria Eng. J. 57 (4), 3727–3735. 10.1016/j.aej.2018.07.018

[B3] Aguilar-Aguilar A. de León-Martínez L. D. Forgionny A. Acelas Soto N. Y. Mendoza S. R. Zárate-Guzmán A. I. (2023). A systematic review on the current situation of emerging pollutants in Mexico: a perspective on policies, regulation, detection, and elimination in water and wastewater. Sci. Total Environ. 905, 167426. 10.1016/j.scitotenv.2023.167426 37774864

[B4] Azizi D. Arif A. Blair D. Dionne J. Filion Y. Ouarda Y. (2022). A comprehensive review on current technologies for removal of endocrine disrupting chemicals from wastewaters. Environ. Res. 207, 112196. 10.1016/j.envres.2021.112196 34634314

[B5] Azzam A. B. El-Sheikh S. M. Geioushy R. A. Salah B. A. El-Dars F. M. Helal A. S. (2019). Facile fabrication of a novel BiPO 4 phase junction with enhanced photocatalytic performance towards aniline blue degradation. RSC Adv. 9 (30), 17246–17253. 10.1039/C9RA02315A 35519846 PMC9064578

[B6] Bunea G. Alexa-Stratulat S. M. Mihai P. Toma I. O. (2023). Use of clay and titanium dioxide nanoparticles in mortar and concrete—a state-of-the-art analysis. Coatings 13 (3), 506. 10.3390/coatings13030506

[B7] Choi Y. C. (2024). Impact of TiO2 powder type on hydration and photocatalytic NOx degradation in cement paste. Results Eng. 24, 103187. 10.1016/J.RINENG.2024.103187

[B8] Cunha D. L. Kuznetsov A. Achete C. A. Machado A. E. da H. Marques M. (2018). Immobilized TiO2 on glass spheres applied to heterogeneous photocatalysis: photoactivity, leaching and regeneration process. PeerJ 2018 (3), e4464. 10.7717/peerj.4464 PMC5844248 29527416

[B9] da Cunha D. L. Camargo da Silva S. M. Bila D. M. da Mota Oliveira J. L. de Novaes Sarcinelli P. Larentis A. L. (2016). Regulation of the synthetic estrogen 17α-ethinylestradiol in water bodies in Europe, the United States, and Brazil. Cad. Saude Publica 32, e00056715. 10.1590/0102-311X00056715 27027456

[B10] Danfá S. Martins R. C. Quina M. J. Gomes J. (2021). Supported tio2 in ceramic materials for the photocatalytic degradation of contaminants of emerging concern in liquid effluents: a review. Molecules 26 (Issue 17), 5363. 10.3390/molecules26175363 34500795 PMC8434047

[B11] Durán A. Monteagudo J. M. San Martín I. Merino S. (2018). Photocatalytic degradation of aniline using an autonomous rotating drum reactor with both solar and UV-C artificial radiation. J. Environ. Manag. 210, 122–130. 10.1016/j.jenvman.2018.01.012 29339330

[B12] Eddy D. R. Puri F. N. Noviyanti A. R. (2015). Synthesis and photocatalytic activity of silica-based sand quartz as the supporting TiO2 photocatalyst. Procedia Chem. 17, 55–58. 10.1016/j.proche.2015.12.132

[B13] Evonik Industries A. G. (2023). TECHNICAL INFORMATION 1243. AEROXIDE®, AERODISP® and AEROPERL® titanium dioxide as photocatalyst. Available online at: https://products.evonik.com/assets/48/71/TI_1243_Titanium_Dioxide_as_Photocatalyst_EN_EN_244871.pdf.

[B14] Feng S. Liu F. Fu X. Peng X. Zhu J. Zeng Q. (2019). Photocatalytic performances and durability of TiO2/cement composites prepared by a smear method for organic wastewater degradation. Ceram. Int. 45 (17), 23061–23069. 10.1016/j.ceramint.2019.07.354

[B15] Galata E. Georgakopoulou E. A. Kassalia M. E. Papadopoulou-Fermeli N. Pavlatou E. A. (2019). Development of smart composites based on doped-TiO2 nanoparticles with visible light anticancer properties. Materials 12 (16), 2589. 10.3390/ma12162589 31416238 PMC6719932

[B16] Gar Alalm M. Tawfik A. Ookawara S. (2016). Enhancement of photocatalytic activity of TiO2 by immobilization on activated carbon for degradation of pharmaceuticals. J. Environ. Chem. Eng. 4 (2), 1929–1937. 10.1016/j.jece.2016.03.023

[B17] Gholami N. Ghasemi B. Anvaripour B. Jorfi S. (2018). Enhanced photocatalytic degradation of furfural and a real wastewater using UVC/TiO2 nanoparticles immobilized on white concrete in a fixed-bed reactor. J. Industrial Eng. Chem. 62, 291–301. 10.1016/j.jiec.2018.01.007

[B18] Gokul Eswaran S. Shahid Afridi P. Vasimalai N. (2023). Effective multi toxic dyes degradation using bio-fabricated silver nanoparticles as a green catalyst. Appl. Biochem. Biotechnol. 195 (6), 3872–3887. 10.1007/s12010-022-03902-y 35435586

[B19] Guayaquil-Sosa J. F. Serrano-Rosales B. Valadés-Pelayo P. J. de Lasa H. (2017). Photocatalytic hydrogen production using mesoporous TiO2 doped with Pt. Appl. Catal. B Environ. 211, 337–348. 10.1016/j.apcatb.2017.04.029

[B20] Guo Z. Huang C. Chen Y. (2020). Experimental study on photocatalytic degradation efficiency of mixed crystal nano-TiO2 concrete. Nanotechnol. Rev. 9 (1), 219–229. 10.1515/NTREV-2020-0019/MACHINEREADABLECITATION/RIS

[B21] Hamad H. Bailón-García E. Morales-Torres S. Carrasco-Marín F. Pérez-Cadenas A. F. Maldonado-Hódar F. J. (2018). Physicochemical properties of new cellulose-TiO2 composites for the removal of water pollutants: developing specific interactions and performances by cellulose functionalization. J. Environ. Chem. Eng. 6 (4), 5032–5041. 10.1016/j.jece.2018.07.043

[B22] Jafari H. Afshar S. (2016). Improved photodegradation of organic contaminants using nano-TiO2 and TiO2-SiO2 deposited on Portland cement concrete blocks. Photochem. Photobiol. 92 (1), 87–101. 10.1111/php.12554 26648581

[B23] Kumar R. Qureshi M. Vishwakarma D. K. Al-Ansari N. Kuriqi A. Elbeltagi A. (2022). A review on emerging water contaminants and the application of sustainable removal technologies. Case Stud. Chem. Environ. Eng. 6, 100219. 10.1016/j.cscee.2022.100219

[B24] López-velázquez K. Cabellos-quiroz J. L. Hoil-canul E. R. (2023). Degradación fotocatalítica de una mezcla de estrógenos en agua utilizando microesferas de BiOBr, 9, 86–95.

[B25] Malakootian M. Nasiri A. Amiri Gharaghani M. (2020). Photocatalytic degradation of ciprofloxacin antibiotic by TiO2 nanoparticles immobilized on a glass plate. Chem. Eng. Commun. 207 (1), 56–72. 10.1080/00986445.2019.1573168

[B26] Meng F. Muhammad Y. Zhou S. Zhu Z. Ye Y. Jiang L. (2022). Preparation of cement mortars incorporating polystyrene-graphene nanosheets and octadecylamine-graphene nanosheets for enhanced mechanical properties. J. Sol-Gel Sci. Technol. 101 (1), 287–298. 10.1007/s10971-021-05677-w

[B27] Mishra U. K. Chandel V. S. Yadav A. K. Gautam A. K. Anand A. D. Varun J. (2024). Synthesis, characterization, and study of photocatalytic degradation of aniline blue dye using copper oxide nanoparticles prepared by Santa Maria feverfew leaf extract. Nanotechnol. Environ. Eng. 9 (3), 473–482. 10.1007/S41204-024-00378-5/FIGURES/10

[B28] Monteserín C. Blanco M. Juarros A. Today A. G.-C. (2024). Solar-assisted stainless-steel TiO2-based coatings for water disinfection and decontamination. Elsevier. Available online at: https://www.sciencedirect.com/science/article/pii/S0920586124001676.

[B29] Muhambihai P. Rama V. Subramaniam P. (2020). Photocatalytic degradation of aniline blue, brilliant green and direct red 80 using NiO/CuO, CuO/ZnO and ZnO/NiO nanocomposites. Environ. Nanotechnol. Monit. and Manag. 14, 100360. 10.1016/J.ENMM.2020.100360

[B30] Pedro-Cedillo L. S. Méndez-Novelo R. I. Hernández-Núñez E. Giácoman-Vallejos G. Bassam A. (2019). Removal of BPA from landfill leachates using fenton-adsorption process. Quimica Nova 42 (4). 10.21577/0100-4042.20170354

[B31] Pennington A. M. Okonmah A. I. Munoz D. T. Tsilomelekis G. Celik F. E. (2018). Changes in polymorph composition in P25-TiO2 during pretreatment analyzed by differential diffuse reflectance spectral analysis. J. Phys. Chem. C 122 (9), 5093–5104. 10.1021/acs.jpcc.7b10449

[B32] Rathi B. S. Kumar P. S. Show P. L. (2021). A review on effective removal of emerging contaminants from aquatic systems: current trends and scope for further research. J. Hazard. Mater. 409, 124413. 10.1016/j.jhazmat.2020.124413 33183841

[B33] Rathi B. S. Kumar P. S. Vo D. V. N. (2021). Critical review on hazardous pollutants in water environment: occurrence, monitoring, fate, removal technologies and risk assessment. Sci. Total Environ. 797, 149134. 10.1016/j.scitotenv.2021.149134 34346357

[B34] Siah W. R. Lintang H. O. Shamsuddin M. Yuliati L. (2016). High photocatalytic activity of mixed anatase-rutile phases on commercial TiO2 nanoparticles. IOP Conf. Ser. Mater. Sci. Eng. 107 (1), 012005. 10.1088/1757-899X/107/1/012005

[B35] Sraw A. Kaur T. Pandey Y. Sobti A. Wanchoo R. K. Toor A. P. (2018). Fixed bed recirculation type photocatalytic reactor with TiO2 immobilized clay beads for the degradation of pesticide polluted water. J. Environ. Chem. Eng. 6 (6), 7035–7043. 10.1016/j.jece.2018.10.062

[B36] Thakurata D. G. Das K. C. Dhar S. S. (2022). Efficient photocatalytic degradation of aniline blue under solar irradiation by ternary cobalt ferrite/graphitic carbon nitride/bentonite nanocomposite. Environ. Sci. Pollut. Res. 29 (23), 34269–34277. 10.1007/s11356-021-18242-3 35037149

[B37] Tobaldi D. M. Pullar R. C. Seabra M. P. Labrincha J. A. (2014). Fully quantitative X-ray characterisation of Evonik Aeroxide TiO2 P25®. Mater. Lett. 122, 345–347. 10.1016/j.matlet.2014.02.055

[B38] Tran M. L. Fu C. C. Chiang L. Y. Hsieh C.Te Liu S. H. Juang R. S. (2020). Immobilization of TiO2and TiO2-GO hybrids onto the surface of acrylic acid-grafted polymeric membranes for pollutant removal: analysis of photocatalytic activity. J. Environ. Chem. Eng. 8 (5), 104422. 10.1016/j.jece.2020.104422

[B39] Ulukaya S. Yoruç A. B. H. Yüzer N. Oktay D. (2017). Material characterization of byzantine period brick masonry walls revealed in Istanbul (Turkey). Period. Polytech. Civ. Eng. 61 (2). 10.3311/PPci.8868

[B40] Vásquez D. Palominos F. Martínez S. (2020). Visible-light photocatalytic degradation of aniline blue by stainless-steel foam coated with tio2 grafted with anthocyanins from a maqui-blackberry system. Catalysts 10 (11), 1245. 10.3390/catal10111245

[B41] Zeng G. You H. Du M. Zhang Y. Ding Y. Xu C. (2021). Enhancement of photocatalytic activity of TiO2 by immobilization on activated carbon for degradation of aquatic naphthalene under sunlight irradiation. Chem. Eng. J. 412, 128498. 10.1016/j.cej.2021.128498

[B42] Zhang C. Chen H. Xue G. Liu Y. Chen S. Jia C. (2021). A critical review of the aniline transformation fate in azo dye wastewater treatment. J. Clean. Prod. 321, 128971. 10.1016/J.JCLEPRO.2021.128971

[B43] Zhang W. Wu Y. Wang J. Liu J. Lu H. Zhai S. (2019). Adsorption of thallium(I) on rutile nano-titanium dioxide and environmental implications. PeerJ 7, e6820. 10.7717/peerj.6820 31143532 PMC6526007

